# Combinatorial Click Chemistry Labeling to Study Live Human Gut-Derived Microbiota Communities

**DOI:** 10.3389/fmicb.2021.750624

**Published:** 2021-10-27

**Authors:** Haitham Hajjo, Neerupma Bhardwaj, Tal Gefen, Naama Geva-Zatorsky

**Affiliations:** ^1^Department of Cell Biology and Cancer Science, Rappaport Faculty of Medicine, Rappaport Technion Integrated Cancer Center (RTICC), Technion-Israel Institute of Technology, Haifa, Israel; ^2^Department of Immunology, Rappaport Faculty of Medicine, Technion-Israel Institute of Technology, Haifa, Israel; ^3^Department of Neuroscience, Rappaport Faculty of Medicine, Technion-Israel Institute of Technology, Haifa, Israel; ^4^CIFAR, MaRS Centre, Toronto, ON, Canada

**Keywords:** metabolic labeling, anaerobic bacteria, *Enterobacteriaceae*, click chemistry, gut microbiome

## Abstract

Gut bacteria were shown to exert pivotal effects on health and disease. However, mechanistic studies of gut bacterial communities are limited due to the lack of technologies for real-time studies on live bacteria. Here, we developed COMBInatorial cliCK-chemistry (COMBICK) labeling on human gut-derived bacteria, both aerobic and anaerobic strains, to enable dynamic tracing of live, heterogeneous bacterial communities on the strain level, including clinical isolates of the *Enterobacteriaceae* family. We further show that COMBICK labeling is applicable on anaerobic bacterial strains directly isolated from stool. In COMBICK, the number of labeled bacteria that can be simultaneously differentiated increases exponentially depending on the availability of fluorophores and machine capabilities. This method allows real-time studies of bacterial communities from a variety of ecosystems, and can significantly advance mechanistic research in the microbiome field.

## Introduction

**FIGURE 1:**
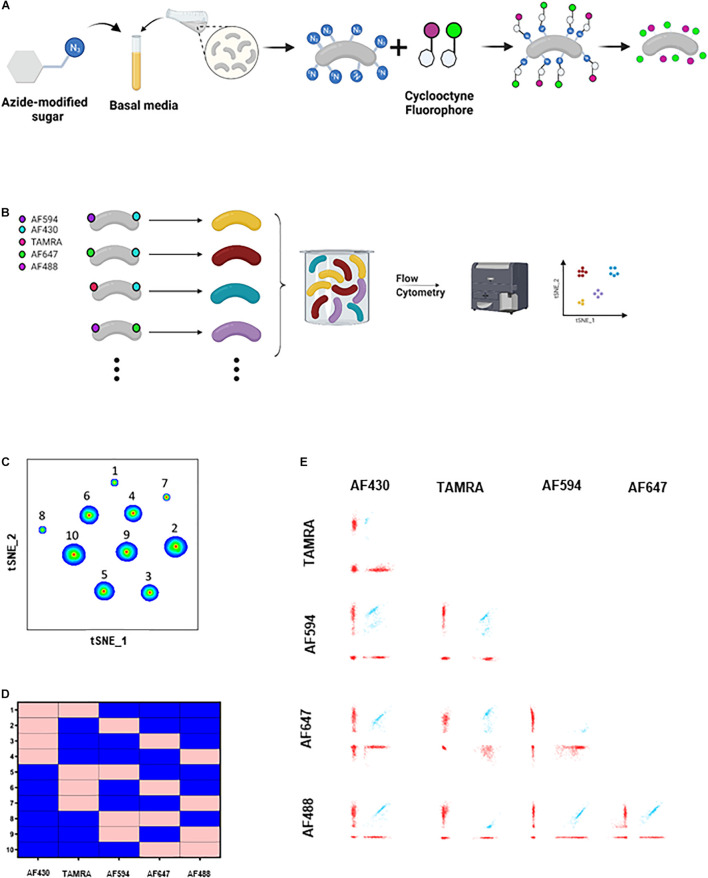
COMBICK dual labeling with 5 fluorophores allows differentiation of up to 10 bacteria at once. **(A)** Schematic representation of the metabolic labeling procedure. Bacteria are grown in the presence of azide-modified sugars and subsequently are incubated with a cyclooctyne tagged fluorophores which covalently react with the azide group turning the bacteria fluorescent. **(B)** Schematic representation of the COMBICK method. Metabolic labeling of each bacteria with a combination of fluorophores results in a unique label that enables the differentiation of specific populations of bacteria in communities. Combinatorically labeled bacteria can be analyzed by downstream applications (e.g., flow cytometry). **(C)** t-SNE plot of flow cytometry analysis of a mixture of *B. longum* bacteria labeled with 10 different dual combinations. **(D)** Matrix demonstrating the dual label of each of the 10 *B. longum* populations identified in panel **(B)** and analyzed by flow cytometry. Pink represents positive labeling and blue negative. **(E)** Flow cytometry dot plots of the 10 different clusters of *B. longum* identified in panel **(C)**.

The human microbiome is one of the fastest growing areas of research in the past two decades (Sommer and Bäckhed, 2013; Gilbert et al., 2018). Many gut bacteria have been shown to modulate physiological functions and pathologies (Gilbert et al., 2016; Elinav et al., 2019; Lynch et al., 2019). Nevertheless, mechanistic understanding is still lacking (Fischbach, 2018; Lawson et al., 2019; Vrancken et al., 2019). Various tools were developed to visualize and detect gut commensals (Valm et al., 2011; Earle et al., 2015; Welch et al., 2016, 2017; Tropini et al., 2017; Whitaker et al., 2017; Sheth et al., 2019). Commonly used fluorescent labeling methods, such as Green Fluorescent Proteins (GFP) and derivatives, require oxygen to mature and fluoresce and hence are inapplicable to live monitoring of gut bacteria in mammals, which are mostly anaerobes. To overcome this challenge, we recently developed a method to fluorescently label live, culturable, and anaerobic gut bacteria with fluorophores that do not require oxygen to mature and fluoresce. This method was based on metabolic oligosaccharide engineering and bioorthogonal click chemistry (Geva-Zatorsky et al., 2015). Briefly, bacterial strains are grown in the presence of azido-modified sugars. During the bacterial growth, these azido-sugars are incorporated in bacterial biomolecules. Then, the bacteria are incubated with fluorescently tagged cyclooctynes, which covalently react with the azide group, turning the bacteria fluorescent (Figure 1A). This method, however, is limited to studying only a few bacteria at a time, while the gut microbiota consists of a plethora of bacterial species residing in functional multi-species communities. Here, we report development of COMBInatorial cliCK-chemistry (COMBICK) labeling of human-derived gut bacteria that enables studying diverse communities of live gut symbionts in their natural environments.

## Materials and Methods

### Bacterial Strains

The bacterial species and strains used are mentioned in Supplementary Tables 1–3. All bacteria were grown in a basal peptone-yeast broth containing (per liter) 5 g of yeast extract, 20 g of protease peptone, 5 g of NaCl, 5 mg of hemin, 0.5 mg of vitamin K1, and 5 g of K_2_HPO_4_. Hemin, vitamin K1, and K_2_HPO_4_ were filtered and supplemented after autoclave.

### Metabolic Labeling of Bacteria

Bacteria were grown overnight at 37°C under anaerobic conditions (80% N_2_, 10% H_2_, 10% CO_2_) in an anaerobic chamber in basal peptone-yeast broth with azido sugars supplemented at a final concentration of 100 μM of each (Click-IT^TM^ GalNAz Metabolic Glycoprotein Labeling Reagent Thermo Fisher Scientific Cat#: C33365, Click-IT^TM^ ManNAz Metabolic Glycoprotein Labeling Reagent Thermo Fisher Scientific Cat#: C33366, Click-IT^TM^ GlcNAz Metabolic Glycoprotein Labeling Reagent Thermo Fisher Scientific Cat#: C33367). Bacteria were washed three times in 1× PBS supplemented with 3% BSA. The final pellet was suspended in 500 μl of 1% BSA in 1× PBS. Fluorophores conjugated to dibenzocyclooctynes (AZDye 405 DBCO Click Chemistry Tools Cat#: 1310, AZDye 430 DBCO Click Chemistry Tools Cat#: 1274, Click-iT Alexa Fluor 488 DIBO alkyne Thermo Fisher Scientific Cat#: C10405, AZDye 594 DBCO Click Chemistry Tools Cat#: 1298, AZDye 647 DBCO Click Chemistry Tools Cat#: 1302, Click-iT TAMRA DIBO alkyne Thermo Fisher Scientific Cat#: C10410) were added at a final concentration of 20 μM each, for single- and dual-fluorophore labeling. For experiments with more than two fluorophores, AZDye 405-DBCO and AZDye 430-DBCO, were added at a final concentration of 100 μM. Tubes were incubated while rocking and protected from light at RT for 5 h, or at 37°C for 1 h. Next, bacteria were washed five times with 3% BSA in 1× PBS followed by a final wash in 1× PBS. Finally, bacteria were resuspended in 1 ml 1× PBS.

### Flow Cytometry

Samples were run on the High Throughput LSR Fortessa, BD BioSciences^®^. Data were processed with Kaluza^®^ and FlowJo^®^ softwares. For each fluorophore, intermediate fluorescence was eliminated, to avoid false-positive and false-negative analysis. For high dimensionality reduction analysis data were processed with FlowJo^®^ software. For each fluorescent channel an analogous binary parameter was derived. The binary parameter equals 1 if the single bacteria was positive for the relevant channel otherwise it equals 0. To discard unlabeled bacteria and bacteria aggregates a new parameter was derived, which equals the sum of all binary parameters. This parameter contains the number of positive fluorophores for each event. According to this parameter, the relevant events were gated. Next, based on the binary parameters t-SNE analyses for dimensionality reduction were done using the FlowJo^®^ software.

### Confocal Microscopy

Zeiss LSM 880 (Carl Zeiss, Jena, Germany) confocal microscope equipped with a GaAsP spectral detector at 8.9 nm lambda channels between 415 and 700 nm and a Plan-Apochromat 63×, 1.4 N.A. objective lens, was used. Fluorophores (AF405-DBCO, AF488-DIBO, AF594-DBCO, AF647-DBCO) were used to label the bacteria. Excitation wavelengths of 633 nm, 561 nm, 488 nm, and 405 nm were employed simultaneously.

### Bacteria Isolation From Stool

Stool pellets were collected from mice colonized with a humanized microbiome. The pellets were resuspended in sterile 1× PBS (100 mg stool: 1 ml sterile 1× PBS) and then centrifuged at 300 *g* for 2 min. The supernatants were then diluted in sterile 1× PBS, plated on BHIS agar plates and grown for 2 days at 37°C under anaerobic conditions (80% N_2_, 10% H_2_, 10% CO_2_) in an anaerobic chamber. Single bacterial colonies were then isolated and grown in basal peptone-yeast broth supplemented with azido modified sugars and labeled using COMBICK as was previously described.

## Results and Discussion

**FIGURE 2:**
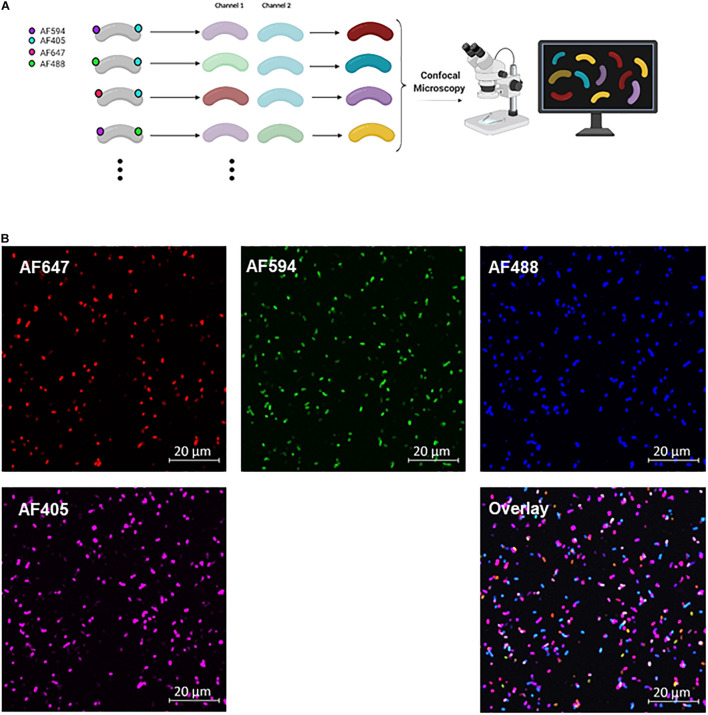
COMBICK dual labeling with 4 fluorophores allows differentiation of up to 6 bacteria at once. **(A)** Schematic representation of the COMBICK method. Metabolic labeling of each bacteria with a combination of fluorophores results in a unique label that enables the differentiation of specific population of bacteria in communities. Combinatorically labeled bacteria can be analyzed by downstream applications (e.g., confocal microscopy). **(B)** Confocal images representing 6 differentially dual-labeled *B. longum* in a community with combinations of the fluorophores AF647, AF594, AF488, and AF405, each channel separately and the overlay of all channels.

In COMBICK we metabolically label each bacteria with a combination of different fluorophores. Each combination allows unique identification of the corresponding bacteria. The number of labeling combinations depends on the number of fluorophores available (“n”), the machine specifications, and the number of fluorophores used to label each bacteria (“k”), and can be calculated by the formula: (nk)=n!k!⁢(n-k)!. For example, with 5 fluorophores available, and each bacteria labeled with a combination of two, 10 different bacteria can be simultaneously differentiated, according to the formula: (52)=5!2!*⁢3!=10. Downstream methods such as flow cytometry and confocal microscopy could be used to differentiate and analyze the labeled bacterial populations (Figures 1B, 2A).

To apply this method, we first chose one bacteria, *Bifidobacterium longum*, and applied 2-color COMBICK labeling using 5 different fluorophores (AF594, AF430, TAMRA, AF647, and AF488) (Figure 1B). This created 10 different populations of *B. longum* that could be differentiated by flow cytometry and analyzed either by manual, classical analysis (Supplementary Figure 1), or by semi-supervised, semi-automatic analysis as applied here (Figures 1C–E). Briefly, for each fluorescent channel we created an analogous binary parameter. The binary parameter equals 1 if the single bacteria was positive for the relevant channel otherwise it equals 0. Then, a new parameter was derived, which contains the number of positive fluorophores for each event. Using this parameter, we could discard unlabeled bacteria and bacteria aggregates such that only relevant events were gated. Next, based on the binary parameters we performed t-SNE analyses for dimensionality reduction to identify bacterial populations according to their fluorophores profile. We confirmed the applicability of COMBICK dual-labeling on 15 phylogenetically diverse human-derived gut commensals (Supplementary Figure 2 and Supplementary Table 1).

Using COMBICK to study bacterial consortia is not restricted to flow cytometry only, but can also be applied with other methods such as confocal microscopy (Figure 2A). By confocal microscopy we could differentiate between 6 different dual-labeled *B. longum* populations using 4 fluorophores (AF405, AF488, AF594, and AF647) (Figure 2B).

**FIGURE 3:**
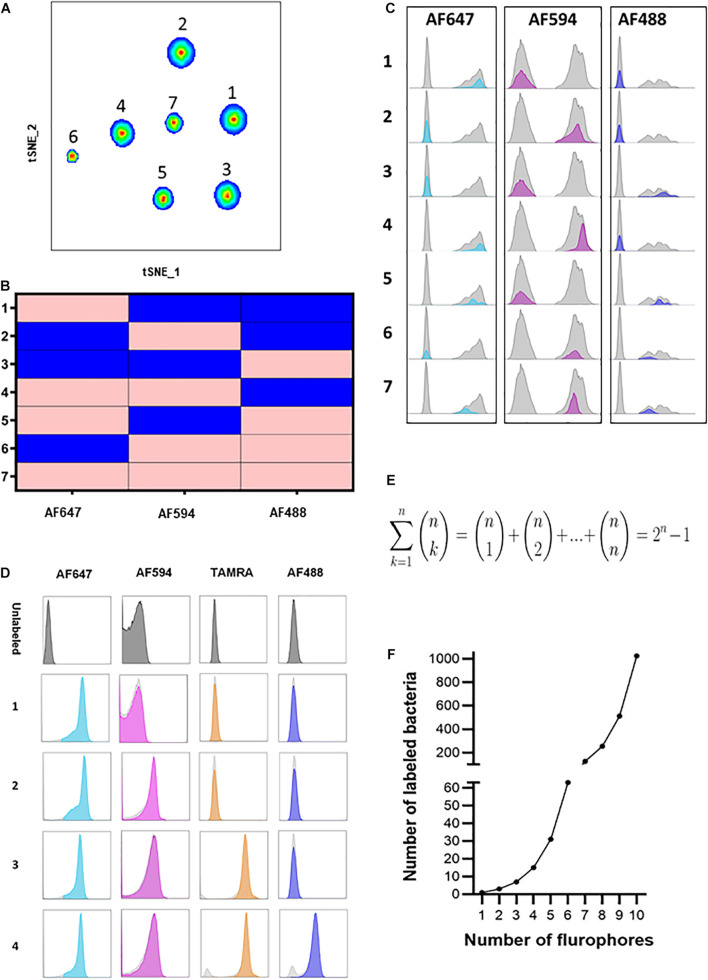
Heterogeneous COMBICK labeling enables analysis of high-order and diverse bacterial consortia. **(A)** Flow cytometry t-SNE plot analysis of 7 different *B. fragilis* strains (YCH46, 1262, ATCC 23745, NCTC 2429, NCTC 9343, 2244, and CL03T00C08, respectively) labeled with 7 different single, dual and triple fluorophore combinations (AF488, AF594, and AF647). **(B)** Matrix demonstrating the single, dual and triple label of each of the 7 different *B. fragilis* strains identified in panel **(A)** and analyzed by flow cytometry. Pink represents positive labeling and blue negative. **(C)** Histograms of the 7 different *B. fragilis* strains analyzed in panel **(A)**. **(D)** Representative flow cytometry histograms of *B. fragilis* labeled with 1, 2, 3, and 4 fluorophores simultaneously. **(E)** Equation representing the number of all possible bacteria labeling combinations with “n” fluorophores. **(F)** Simulation of the equation described in panel **(E)**.

Recent studies have demonstrated the importance of the microbiome at the strain level (Yang et al., 2020; Hajjo and Geva-Zatorsky, 2021). These studies emphasized functional differences of different strains of the same species, albeit their genomes are largely similar. However, technologies that enable studying different strains in parallel are limited. COMBICK enables us to differentiate between several strains of the same species simultaneously. To validate this, we applied COMBICK on several different strains of *B. fragilis* in a multi-strain community (Supplementary Table 2). To do so, we further developed COMBICK to enable labeling of each bacteria with 1, 2, or 3 fluorophores simultaneously. Each strain was labeled with a unique combination (all possible combinations with these 3 fluorophores: single-, dual-, or triple-labeled). This enabled us to differentiate between 7 different strains of *B. fragilis* at once using merely 3 fluorophores, utilizing all possible combinations simultaneously (Figures 3A–C).

We further developed COMBICK to enable high-order-fluorophore labeling. We could successfully label single bacteria with combinations of 4 distinct fluorophores simultaneously (Figure 3D). Theoretically, with ‘n’ fluorophores available, COMBICK enables labeling of bacterial populations with combinations of 1, 2, 3 … and up to ‘n’ fluorophores simultaneously. The major limit is the available fluorophores, and machine specifications (i.e., the number of fluorophores the microscopes or flow cytometers can simultaneously detect). Hence, the total number of distinct bacteria that could be differentiated by COMBICK is 2*^n^*−1 (Figures 3E,F). The number of bacteria that could be labeled simultaneously grows exponentially depending on the number fluorophores used according to the formula: (n1)+(n2)+(n3)+⋯⁢(nn)=∑k=1n(nk)=2n-1. The semi-supervised analysis we applied here, enables semi-automatic analyses of such large consortia.

**FIGURE 4:**
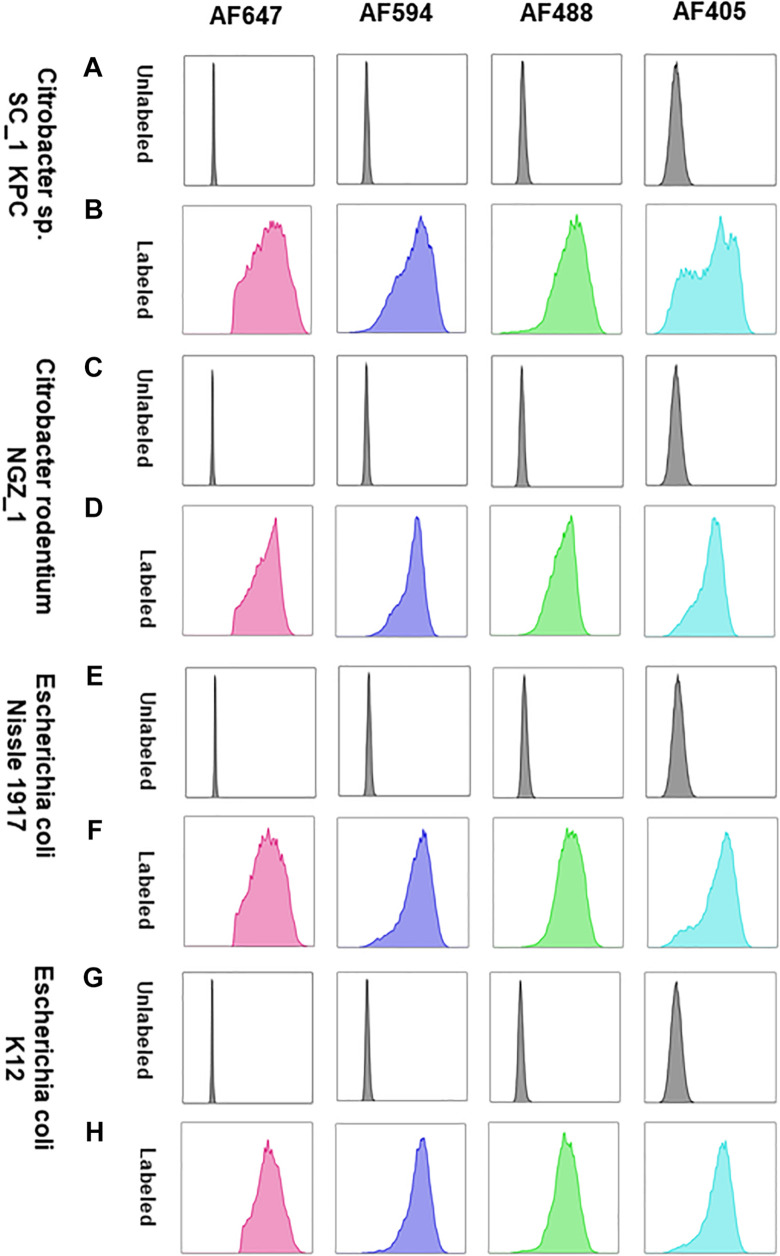
COMBICK enables multi-fluorophore labeling of different *Enterobacteriaceae* species using flow cytometry. Labeling of different *Enterobacteriaceae* species with several fluorophores simultaneously (AF647, AF4594, AF488, and AF405). Each column represents a different fluorophore channel. **(A,C,E,G)** Flow cytometry histograms of the unlabeled *Enterobacteriaceae* species. **(B,D,F,H)** Flow cytometry histograms of the labeled *Enterobacteriaceae* species with 4 fluorophores simultaneously.

**FIGURE 5:**
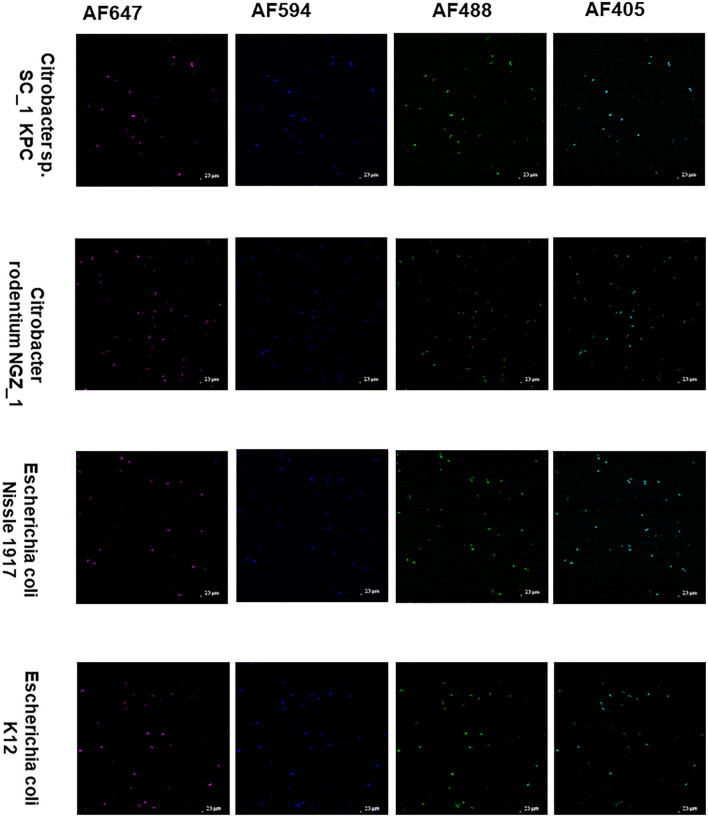
COMBICK enables multi-fluorophore labeling of different *Enterobacteriaceae* species using confocal microscopy. Labeling of different *Enterobacteriaceae* species with several fluorophores simultaneously (AF647, AF4594, AF488, and AF405). Each column represents a different fluorophore channel. Confocal images representing labeled *Enterobacteriaceae* species with 4 fluorophores simultaneously.

The *Enterobacteriaceae* family of bacteria are primary colonizers of mammalian intestines consisting of both symbionts and pathobionts that can cause intestinal diseases (Kelly et al., 2017). Antibiotic resistant *Enterobacteriaceae* are an arising global health hazard due to their increasing prevalence and limited effective eradication treatments. In many cases, gut colonizing strains are the source for *Enterobacteriaceae* diseases (Dickstein et al., 2016; Bar-Yoseph et al., 2021). Thus, developing methods that enable studying and understanding *Enterobacteriaceae* mechanisms of colonization, dynamic interactions with other gut microbiota members and their effects on the host in health and disease is timely and clinical relevant. Therefore, we aimed to validate COMBICK on a panel of *Enterobacteriaceae* species, both lab strains and clinical isolates, including antibiotic resistant isolates (Supplementary Table 3). Our panel included bacteria from *Citrobacter*, *Klebsiella* and *Escherichia* genera. We succeeded to label different strains of *Escherichia coli*, *Citrobacter rodentium*, and more (Figures 4, 5 and Supplementary Figures 3, 4). Among the *Enterobacteriaceae* genera that we tested, *Klebsiella* was the one that was inefficiently labeled (Supplementary Figure 5).

**FIGURE 6:**
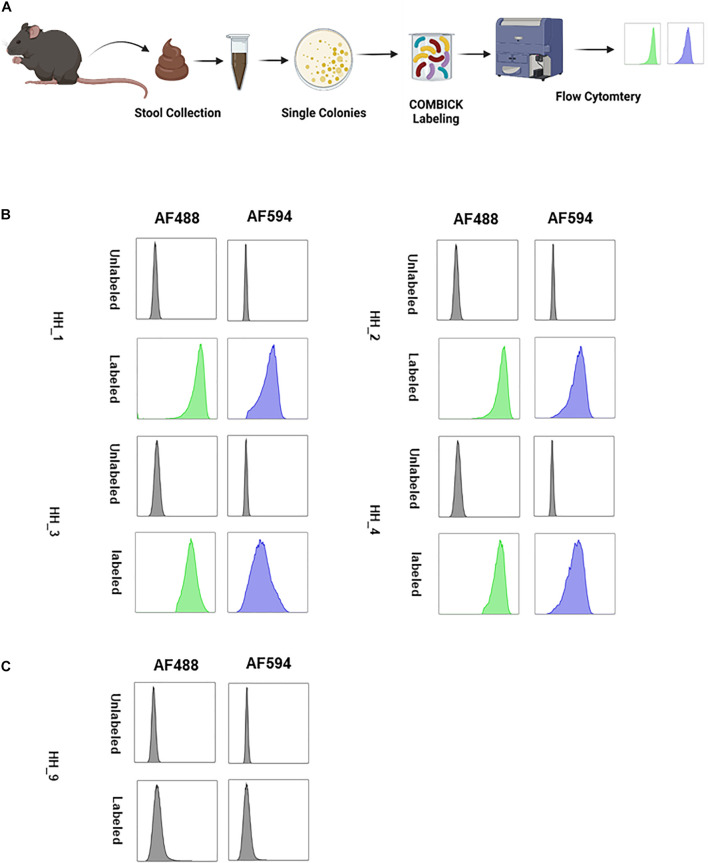
COMBICK enables labeling anaerobic bacteria isolated directly from stool. Labeling live anaerobic bacteria directly isolated from stool. **(A)** Illustration of the experiment scheme. Single bacteria colonies were isolated from stool, labeled by COMBICK and analyzed by flow cytometry. The bacterial isolates were named HH_#, (# for sequential arbitrary numbers). **(B)** Flow cytometry histograms of the isolated bacteria labeled by COMBICK. Each column represents a different fluorophore channel. The upper row of each bacteria represents the unlabeled control, and the lower row represents the bacteria labeled with both fluorophores. **(C)** Flow cytometry histograms of the isolated bacteria that was not successfully labeled by COMBICK. Each column represents a different fluorophore channel. The upper row represents the unlabeled control, and the lower row the bacteria after applying COMBICK labeling.

Lastly, we applied COMBICK on anaerobic bacteria directly isolated from stool. The bacteria were isolated from the stool of “humanized” mice (i.e., mice colonized with a human microbiota), and dual-labeled by COMBICK using AF488 and AF594. Out of 11 isolates 10 were successfully labeled and only one was not (Figure 6 and Supplementary Figure 6).

To summarize, this method is relatively simple, fast, and applicable to bacteria from across phyla and ecosystems, both lab strains and clinical isolates. The synergism between multi-combinatorial labeling and semi-supervised analysis makes COMBICK applicable to study and differentiate mixed populations of live bacteria, including multi-strains consortia. Furthermore, COMBICK allows future studies on microbial colonization, population dynamics, microbe-microbe, microbe-host, and bacteria-phage interactions, as well as analyses of individual bacterial populations in the context of communities in their natural environments. The method presented here opens a window for real-time studies on high-order, diverse gut bacterial communities – commensals, symbionts and pathobionts (i.e., *Enterobacteriaceae*). As such, we believe this method is timely and can significantly facilitate mechanistic and translational research of the gut microbiota.

## Data Availability Statement

The raw data supporting the conclusions of this article will be made available by the authors, without undue reservation.

## Ethics Statement

Ethical review and approval was not required for the animal study because in the experiments involving mice we only collected stool from the mice and did not conduct experiments on them.

## Author Contributions

HH, NB, and TG conceived the project, analyzed the data, interpreted the results, and wrote the manuscript. NG-Z conceived the project, interpreted the results, and wrote the manuscript. All authors contributed to the article and approved the submitted version.

## Conflict of Interest

The authors declare that the research was conducted in the absence of any commercial or financial relationships that could be construed as a potential conflict of interest.

## Publisher’s Note

All claims expressed in this article are solely those of the authors and do not necessarily represent those of their affiliated organizations, or those of the publisher, the editors and the reviewers. Any product that may be evaluated in this article, or claim that may be made by its manufacturer, is not guaranteed or endorsed by the publisher.

## Supplementary Material

Supplementary Figure 1 Classical analysis of a mixture of *B. longum* labeled bacteria with 10 different dual combinations. Flow cytometry dot plots representing *B. longum* dual-labeled with 10 different combinations of fluorophores. Differentiation of a mixture 10 different dual-labeled *B. longum* using classical analysis.

Supplementary Figure 2 COMBICK is applicable on phylogenetically diverse human gut-derived commensals. Flow cytometry histogram plots representing labeling of 15 commensal bacteria, each labeled with two fluorophores (green: AF488, red: AF647). Bacterial strains (1–15) are listed in Supplementary Table 1.

Supplementary Figure 3 COMBICK enables multi-fluorophore labeling of *Enterobacteriaceae* species using flow cytometry Labeling of an additional *Enterobacteriaceae* species with several fluorophores simultaneously (AF647, AF4594, AF488 and AF405). Each column represents a different fluorophore channel. **(A)** Flow cytometry histograms of the unlabeled bacteria. **(B)** Flow cytometry histograms of the labeled bacteria with 4 fluorophores simultaneously.

Supplementary Figure 4 COMBICK enables multi-fluorophore labeling of different *Enterobacteriaceae* species using confocal microscopy. Labeling of an *Enterobacteriaceae* species with several fluorophores simultaneously (AF647, AF4594, AF488, and AF405). Each column represents a different fluorophore channel. Confocal images representing the labeled *Enterobacteriaceae* species with four fluorophores simultaneously.

Supplementary Figure 5 COMBICK labeling is not applicable on different Klebsiella species. Labeling of different Klebsiella species with several fluorophores simultaneously (AF647, AF4594, AF488, and AF405). Each column represents a different fluorophore channel. **(A,C,E)** Histograms of flow cytometry analysis of unlabeled Klebsiella species. **(B,D,F)** Flow cytometry histograms of Klebsiella species labeled using COMBICK.

Supplementary Figure 6 COMBICK enables labeling anaerobic bacteria isolated directly from stool. Labeling live anaerobic bacteria directly isolated from stool. Flow cytometry histograms of the isolated bacteria labeled by COMBICK. Each column represents a different fluorophore channel. The upper row of each bacteria represents the unlabeled control, and the lower row the bacteria labeled with both fluorophores. The bacterial isolates were named HH_#, (# for sequential arbitrary numbers).
