# Supplementary figures and images for: Combinatorial Click Chemistry Labeling to Study Live Human Gut-Derived Microbiota Communities
Source: Front Microbiol. 2021 Oct 27;12:750624. doi: 10.3389/fmicb.2021.750624 (PMC8579052; doi:10.3389/fmicb.2021.750624)

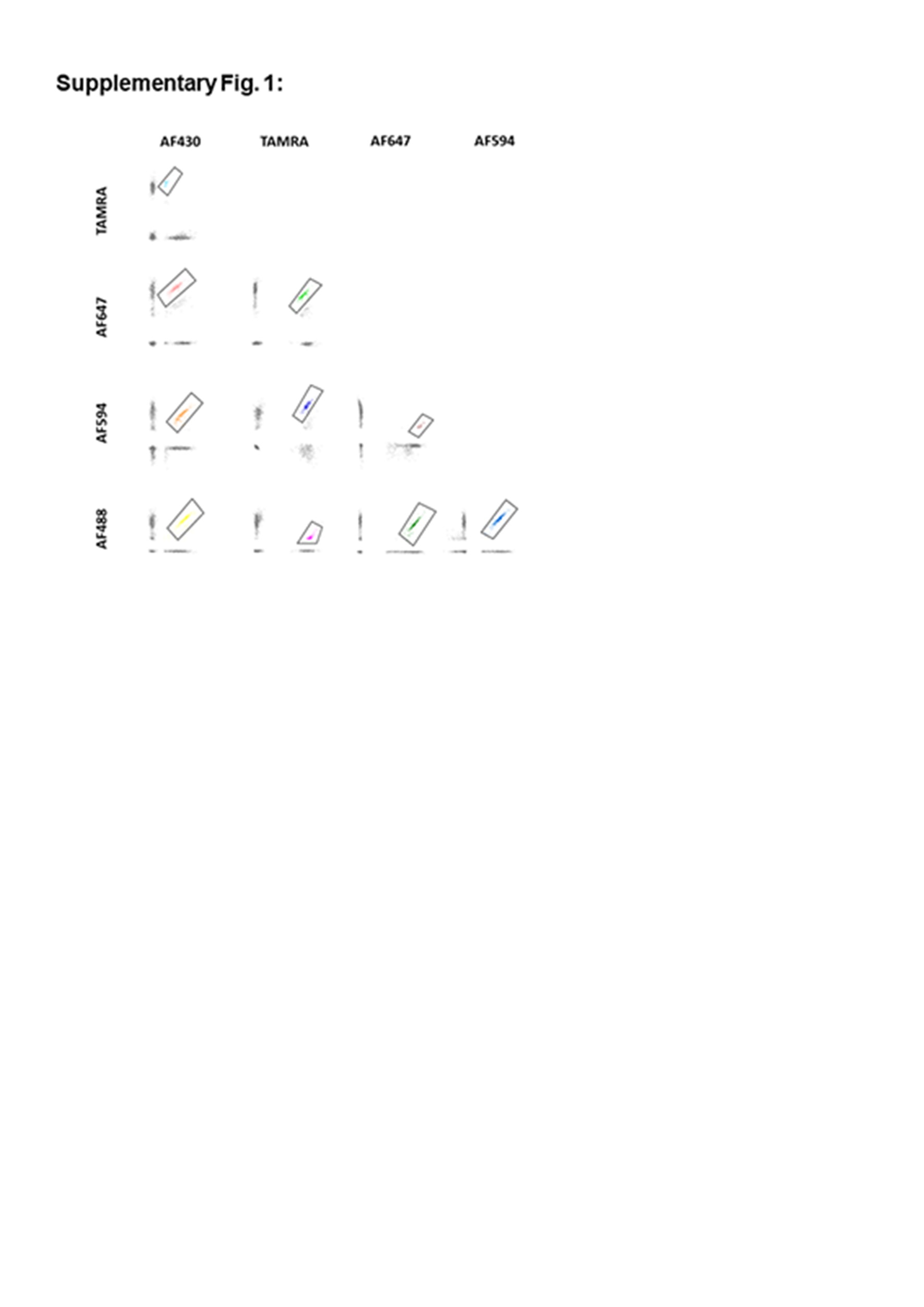

Supplement: Supplementary Figure 1 — Classical analysis of a mixture of B. longum labeled bacteria with 10 different dual combinations. Flow cytometry dot plots representing B. longum dual-labeled with 10 different combinations of fluorophores. Differentiation of a mixture 10 different dual-labeled B. longum using classical analysis. [file Image_1.TIF]

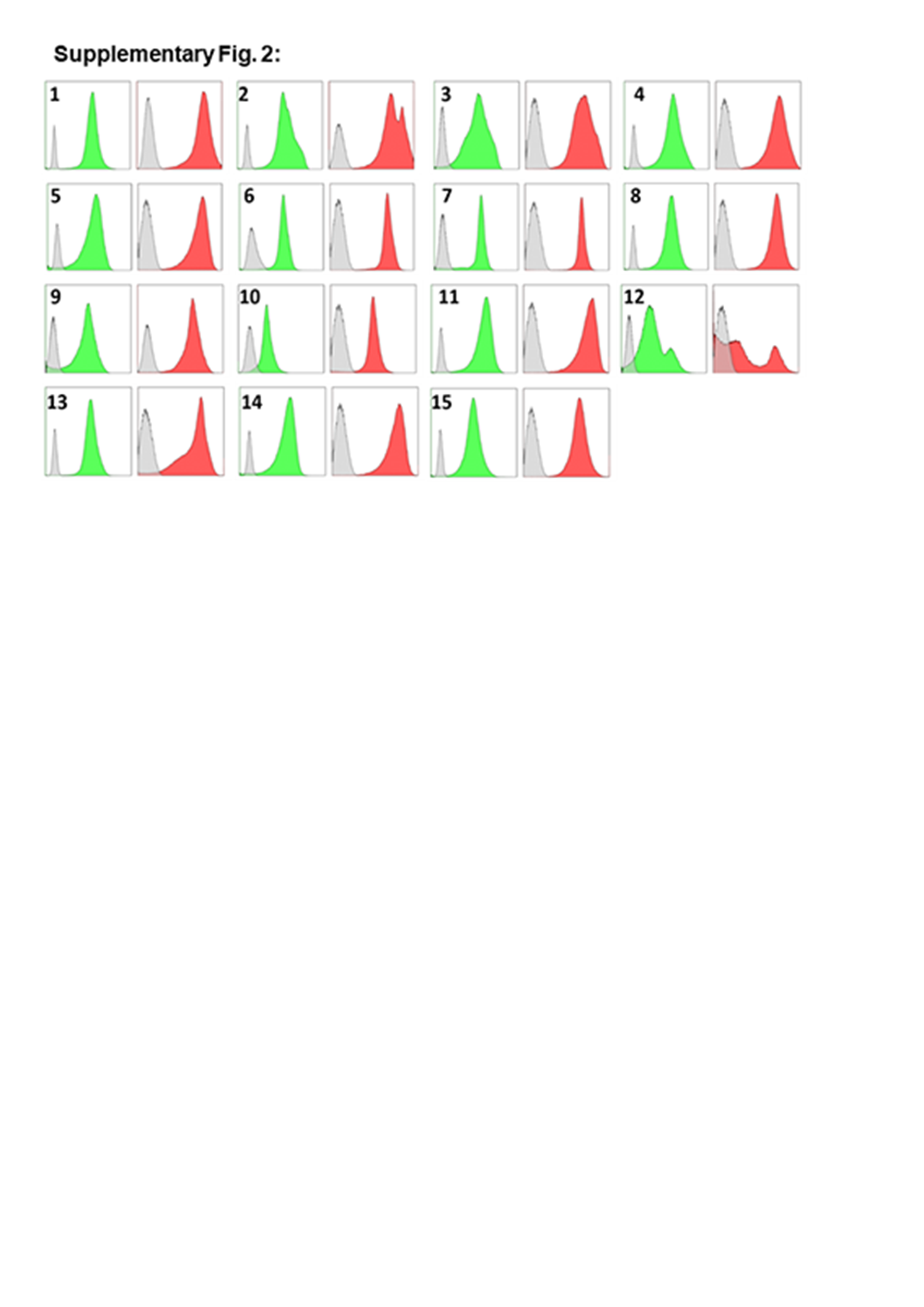

Supplement: Supplementary Figure 2 — COMBICK is applicable on phylogenetically diverse human gut-derived commensals. Flow cytometry histogram plots representing labeling of 15 commensal bacteria, each labeled with two fluorophores (green: AF488, red: AF647). Bacterial strains (1–15) are listed in Supplementary Table 1. [file Image_2.TIF]

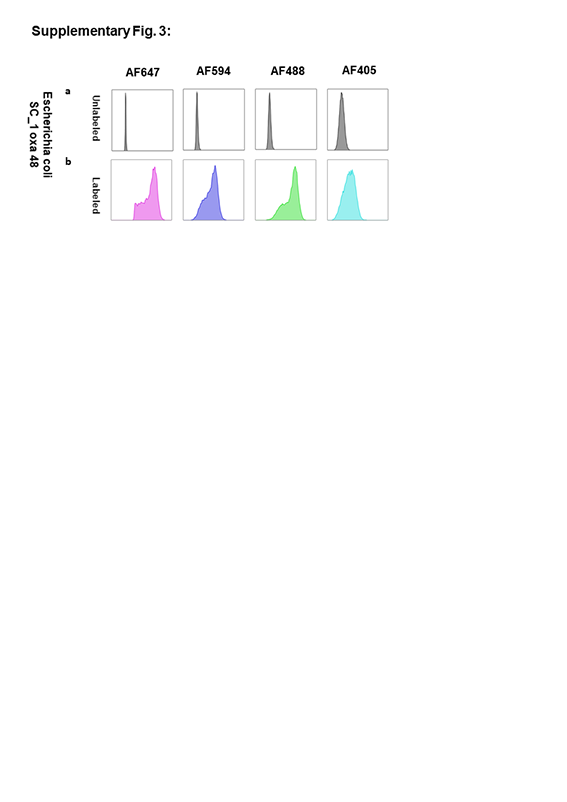

Supplement: Supplementary Figure 3 — COMBICK enables multi-fluorophore labeling of Enterobacteriaceae species using flow cytometry Labeling of an additional Enterobacteriaceae species with several fluorophores simultaneously (AF647, AF4594, AF488 and AF405). Each column represents a different fluorophore channel. (A) Flow cytometry histograms of the unlabeled bacteria. (B) Flow cytometry histograms of the labeled bacteria with 4 fluorophores simultaneously. [file Image_3.TIF]

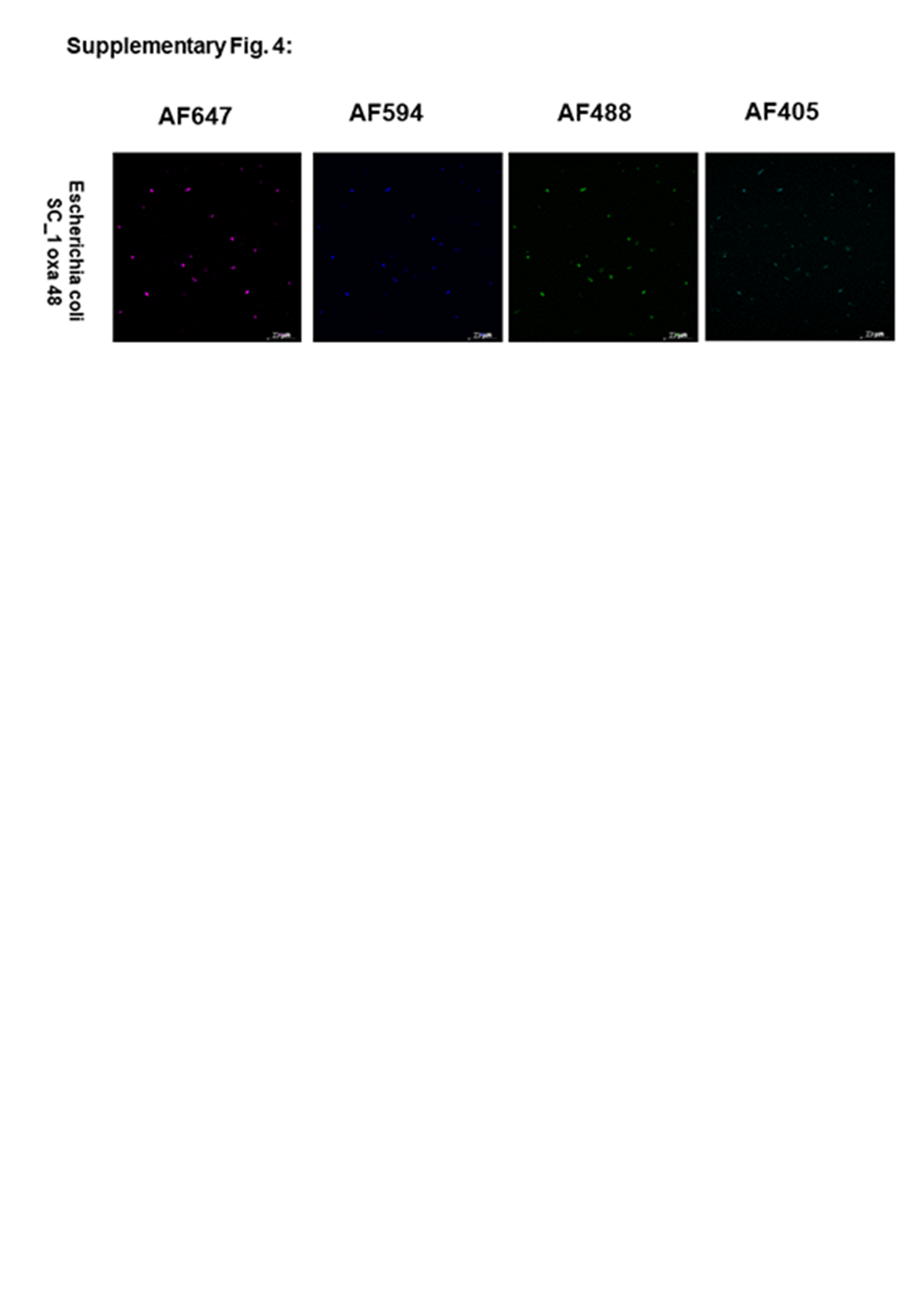

Supplement: Supplementary Figure 4 — COMBICK enables multi-fluorophore labeling of different Enterobacteriaceae species using confocal microscopy. Labeling of an Enterobacteriaceae species with several fluorophores simultaneously (AF647, AF4594, AF488, and AF405). Each column represents a different fluorophore channel. Confocal images representing the labeled Enterobacteriaceae species with four fluorophores simultaneously. [file Image_4.TIF]

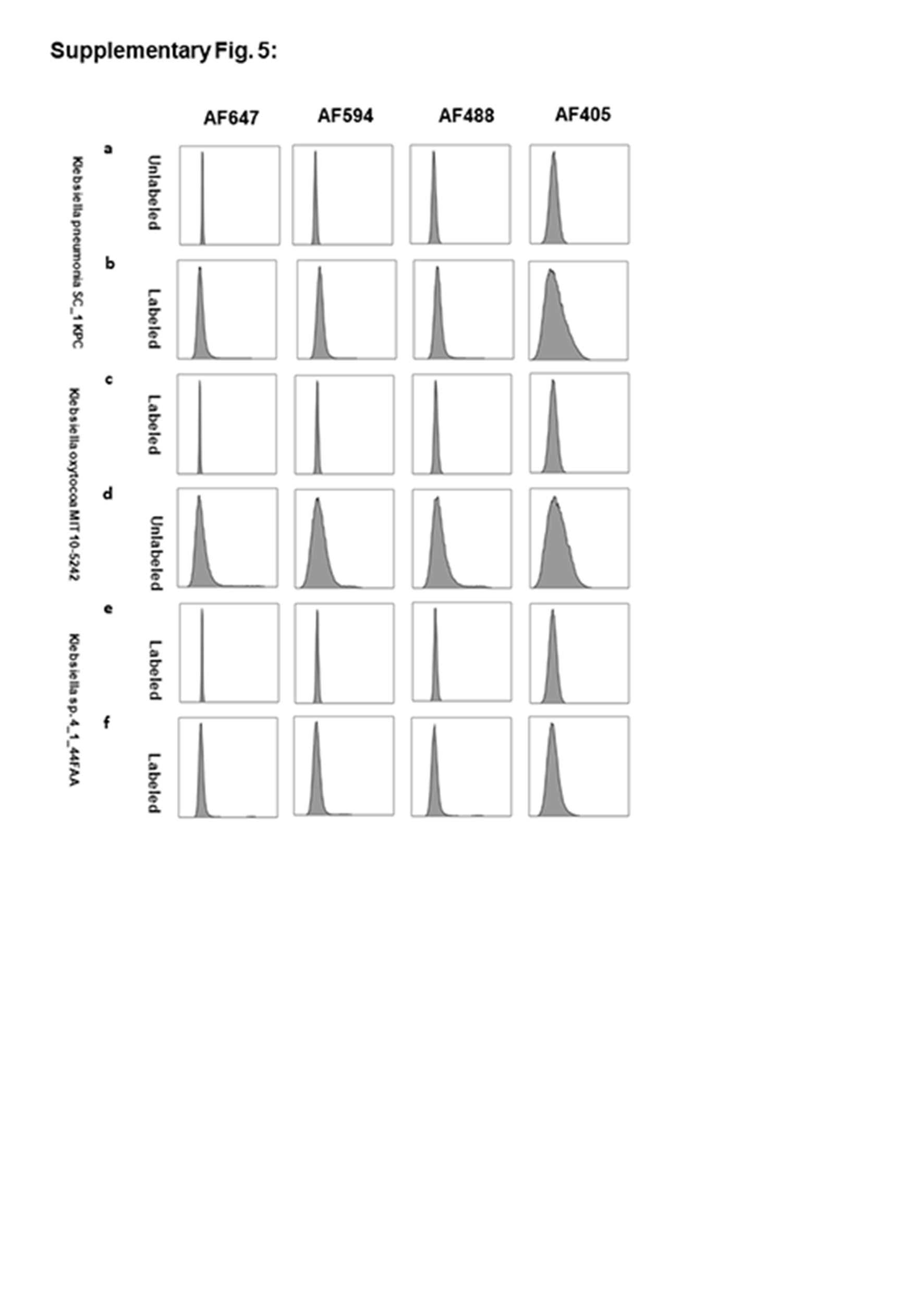

Supplement: Supplementary Figure 5 — COMBICK labeling is not applicable on different Klebsiella species. Labeling of different Klebsiella species with several fluorophores simultaneously (AF647, AF4594, AF488, and AF405). Each column represents a different fluorophore channel. (A,C,E) Histograms of flow cytometry analysis of unlabeled Klebsiella species. (B,D,F) Flow cytometry histograms of Klebsiella species labeled using COMBICK. [file Image_5.TIF]

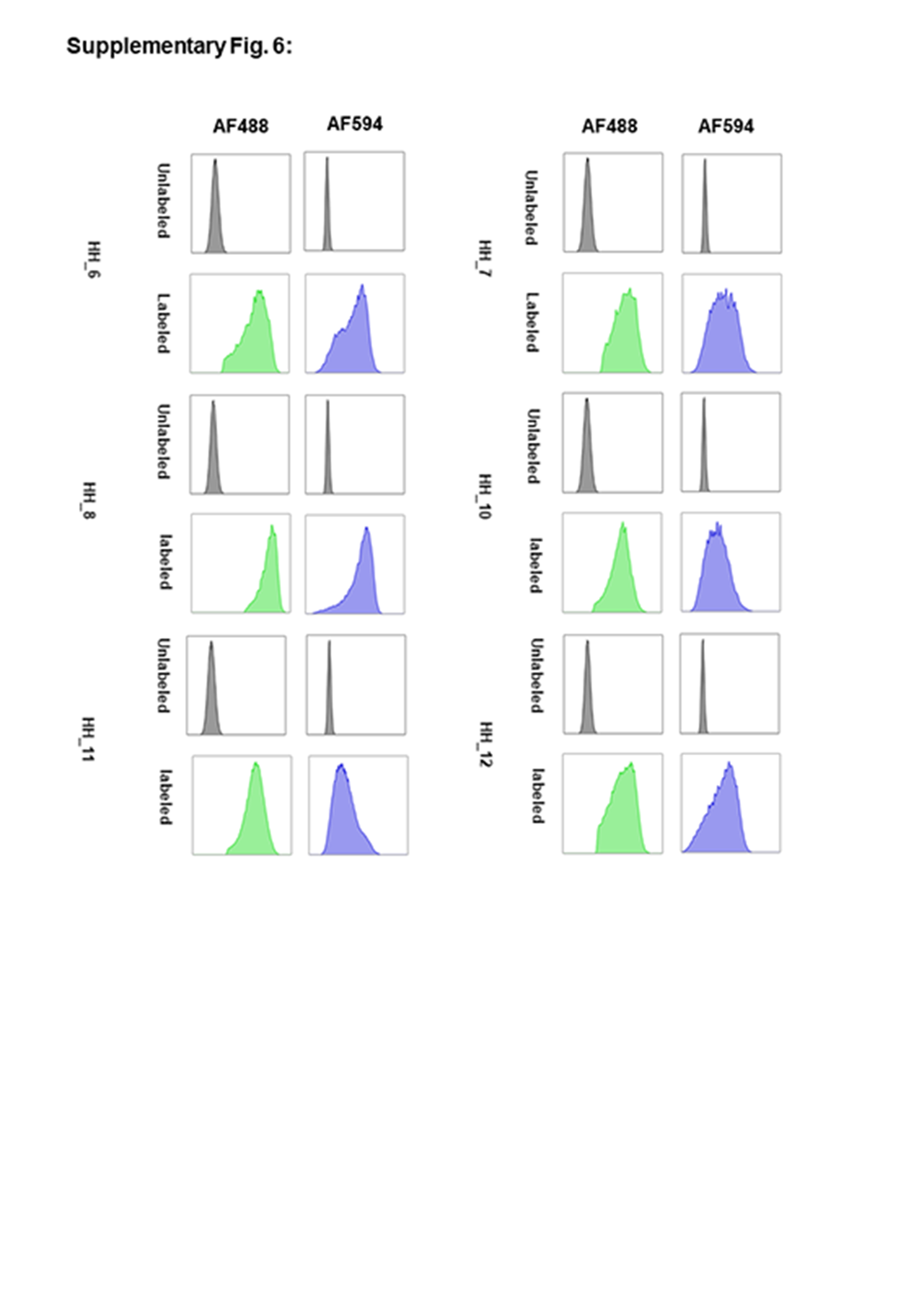

Supplement: Supplementary Figure 6 — COMBICK enables labeling anaerobic bacteria isolated directly from stool. Labeling live anaerobic bacteria directly isolated from stool. Flow cytometry histograms of the isolated bacteria labeled by COMBICK. Each column represents a different fluorophore channel. The upper row of each bacteria represents the unlabeled control, and the lower row the bacteria labeled with both fluorophores. The bacterial isolates were named HH_#, (# for sequential arbitrary numbers). [file Image_6.TIF]
