# Supplementary material for: Combinatorial Click Chemistry Labeling to Study Live Human Gut-Derived Microbiota Communities
Source: Front Microbiol. 2021 Oct 27;12:750624. doi: 10.3389/fmicb.2021.750624 (PMC8579052; doi:10.3389/fmicb.2021.750624)
Supplement: Supplementary file 7 [file Table_1.PDF]

Supplementary Table 1

Phylum	Class	Order	Family	Genus	Species	Strain
Bacteroidetes	Bacteroidia	Bacteroidales	Bacteroidaceae	Bacteroides	Bacteroides fragilis	CL05T00C42
Bacteroidetes	Bacteroidia	Bacteroidales	Bacteroidaceae	Bacteroides	Bacteroides fragilis	CL03T00C08
Bacteroidetes	Bacteroidia	Bacteroidales	Bacteroidaceae	Bacteroides	Bacteroides fragilis	NCTC 2429
Bacteroidetes	Bacteroidia	Bacteroidales	Bacteroidaceae	Bacteroides	Bacteroides fragilis	ATCC 23745
Bacteroidetes	Bacteroidia	Bacteroidales	Bacteroidaceae	Bacteroides	Bacteroides fragilis	1262
Fusobacteria	Fusobacteriia	Fusobacteriales	Fusobacteriaceae	Fusobacterium	Fusobacterium varium	AO27
Proteobacteria	Epsilonproteobacteria	Campylobacterales	Campylobacteraceae	Campylobacter	Campylobacter jejuni	AS-84-79
Bacteroidetes	Bacteroidia	Bacteroidales	Bacteroidaceae	Bacteroides	Bacteroides uniformis	ATCC 8492
Actinobacteria	Actinobacteria	Propionibacteriales	Propionibacteriaceae	Propionibacteriu	Propionibacterium granulosum	AO42
Fusobacteria	Fusobacteriia	Fusobacteriales	Fusobacteriaceae	Fusobacterium	Fusobacterium varium	AO16
Bacteroidetes	Bacteroidia	Bacteroidales	Bacteroidaceae	Bacteroides	Bacteroides fragilis	NCTC 9343
Firmicutes	Clostridia	Clostridiales	Clostridiaceae	Clostridium	Clostridium perfringens	ATCC 13124
Bacteroidetes	Bacteroidia	Bacteroidales	Bacteroidaceae	Bacteroides	Bacteroides thetaiotaomicron	ATCC 29148
Bacteroidetes	Bacteroidia	Bacteroidales	Bacteroidaceae	Bacteroides	Bacteroides caccae	AO1
Bacteroidetes	Bacteroidia	Bacteroidales	Bacteroidaceae	Bacteroides	Bacteroides ovatus	ATCC 8483
