# Supplementary material for: Combinatorial Click Chemistry Labeling to Study Live Human Gut-Derived Microbiota Communities
Source: Front Microbiol. 2021 Oct 27;12:750624. doi: 10.3389/fmicb.2021.750624 (PMC8579052; doi:10.3389/fmicb.2021.750624)
Supplement: Supplementary file 8 [file Table_2.PDF]

Supplementary Table 2

Phylum	Class	Order	Family	Genus	Species	Strain
Bacteroidetes	Bacteroidia	Bacteroidales	Bacteroidaceae	Bacteroides	Bacteroides fragilis	YCH46
Bacteroidetes	Bacteroidia	Bacteroidales	Bacteroidaceae	Bacteroides	Bacteroides fragilis	1262
Bacteroidetes	Bacteroidia	Bacteroidales	Bacteroidaceae	Bacteroides	Bacteroides fragilis	ATCC 23745
Bacteroidetes	Bacteroidia	Bacteroidales	Bacteroidaceae	Bacteroides	Bacteroides fragilis	NCTC 2429
Bacteroidetes	Bacteroidia	Bacteroidales	Bacteroidaceae	Bacteroides	Bacteroides fragilis	NCTC 9343
Bacteroidetes	Bacteroidia	Bacteroidales	Bacteroidaceae	Bacteroides	Bacteroides fragilis	2244
Bacteroidetes	Bacteroidia	Bacteroidales	Bacteroidaceae	Bacteroides	Bacteroides fragilis	CL03T00C08
