# Supplementary material for: Combinatorial Click Chemistry Labeling to Study Live Human Gut-Derived Microbiota Communities
Source: Front Microbiol. 2021 Oct 27;12:750624. doi: 10.3389/fmicb.2021.750624 (PMC8579052; doi:10.3389/fmicb.2021.750624)
Supplement: Supplementary file 9 [file Table_3.PDF]

Supplementary Table 3

Class	Order	Family	Genus	Species	Strain	Comments
Gammaproteobacteria	Enterobacterales	Enterobacteriaceae	Klebsiella	Klebsiella oxytoca	MIT 10-5242	
Gammaproteobacteria	Enterobacterales	Enterobacteriaceae	Klebsiella	Klebsiella pneumoniae	SC_1	clinical isolate, KPC resistance
Gammaproteobacteria	Enterobacterales	Enterobacteriaceae	Klebsiella	Klebsiella sp.	4_1_44FAA	
Gammaproteobacteria	Enterobacterales	Enterobacteriaceae	Citrobacter	Citrobacter rodentium	NGZ_1	
Gammaproteobacteria	Enterobacterales	Enterobacteriaceae	Escherichia	Escherichia coli	Nissle 1917	
Gammaproteobacteria	Enterobacterales	Enterobacteriaceae	Escherichia	Escherichia coli	K-12	
Gammaproteobacteria	Enterobacterales	Enterobacteriaceae	Citrobacter	Citrobacter sp.	SC_1	clinical isolate, KPC resistance
Gammaproteobacteria	Enterobacterales	Enterobacteriaceae	Escherichia	Escherichia coli	SC_1	clinical isolate, oxa48 resistance
